# Sinking Rate and Community Structures of Autumn Phytoplankton Responses to Mesoscale Physical Processes in the Western South China Sea

**DOI:** 10.3389/fmicb.2021.777473

**Published:** 2021-12-06

**Authors:** Yingjie Mao, Xiaoqian Li, Guicheng Zhang, Yan Liao, Gang Qian, Jun Sun

**Affiliations:** ^1^College of Marine Science and Technology, China University of Geosciences (Wuhan), Wuhan, China; ^2^Research Centre for Indian Ocean Ecosystem, Tianjin University of Science and Technology, Tianjin, China

**Keywords:** phytoplankton, community structure, sinking rate, South China Sea, mesoscale physical processes

## Abstract

To examine the influence of mesoscale eddy on the natural phytoplankton community and its sinking rate changes, a comprehensive investigation cruise was carried out in the western South China Sea in autumn 2016. A total of 108 phytoplankton species were found, which belong to 54 phytoplankton genera; most of them were dominated by Dinophyta (54 genera), followed by Bacillariophyta (50 genera), Cyanophyta (3 genera), and Chrysophyta (1 genus). Bacillariophyta and Dinophyta were the main phytoplankton communities in the investigated sea area. The sinking rate of phytoplankton ranged from 0.12 to 3.17 m day^–1^, determined by the SETCOL method. The highest phytoplankton sinking rate was found in the 200-m water layer, followed by the DCM layer. No significant correlation was found between phytoplankton sinking rates and most of the environmental parameters during this cruise. At a similar time, we have carried out the estimation of carbon flux in the investigated sea area by using the sinking rate of phytoplankton, which showed that the carbon flux ranged from 2.41 × 10^–6^ to 0.006 mg C m^–2^ day^–1^; in addition, the maximum was at the 200-m layer. Phytoplankton community and sinking rate were significantly affected by the mesoscale eddy processes. The cold eddy could affect the community distribution of diatom and dinoflagellate, and the upwelling mainly affects the community of dinoflagellate. Both of them could contribute to a higher sedimentation rate of phytoplankton in the surface and DCM layers. Warm eddy could reduce the abundance of phytoplankton in the surface layer; simultaneously, the sinking rate of phytoplankton in the shallow water layer above 100 m is also reduced. These results can fill in the knowledge gap of mesoscale eddy processes in the study of phytoplankton community change and sinking rate; furthermore, it can provide insights into phytoplankton carbon and its implementation in further carbon sink.

## Introduction

The largest ecosystem on earth is the marine ecosystem. The sea surface occupies nearly 71% of the earth’s surface, and seawater accounts for about 97.5% of the earth’s water (Peng, 2000). As a component and regulator in marine ecosystem, phytoplankton play an important role in the global carbon cycle (Sun, 2011). Phytoplankton can trap approximately 3 to 5 billion tons of carbon per year, which accounts for 40 to 50% of the world’s total primary productivity (Raymont, 2014). Although marine phytoplankton are tiny individuals and only account for 1% of the total global plant life, they fix as much net carbon dioxide as land plants, and perform nearly half of photosynthetic carbon fixation and half of the oxygen production (Hutchins and Boyd, 2016). In terms of spatial and temporal distribution, phytoplankton are more widely distributed and can respond more rapidly to environmental changes and, thus, are important to the global carbon cycle (Behrenfeld, 2014; Behrenfeld et al., 2017).

The South China Sea (SCS) is one of the largest semi-enclosed marginal sea, located in the tropical and subtropical regions of Southern Asia (Su, 2004; Guan and Yuan, 2006). The SCS, which is located in the East Asian monsoon region, has its own circulation structure, in which the circulation brought by the monsoon can affect the upper waters in the sea area (Fang et al., 2002). Affected by the monsoon and complex terrain, some mesoscale physical processes are expected to form in this sea area, such as mesoscale eddies, upwellings, and riverine input (Xue et al., 2001; Wu et al., 2002; Wu and Li, 2003; Lu et al., 2010). In the SCS, the southwest monsoon prevails in summer and autumn, and the northeast wind prevails in winter (Su, 2004; Guan and Yuan, 2006). Due to the monsoon, mesoscale physical processes can significantly affect the growth and distribution of phytoplankton by altering the marine environment (Liao et al., 1999; Shiah et al., 2000; Wu et al., 2016). The western South China Sea (wSCS) is one of the main regions with active mesoscale physical processes (Zhuang et al., 2010). In the wSCS, one study reported that the total chlorophyll *a* (Chl-*a*) in the warm eddy area increased significantly while that in the cold eddy area did not change significantly. In the meantime, the contribution of Haptophyta decreased while that of *Prochlorococcus* and *Synechococcus* increased in the warm eddy area. In the cold eddy area, the Bacillariophyta contribution increased, while the *Synechococcus* contribution decreased (Zhong et al., 2013). Another study confirmed that compared with cyclonic vortices, anticyclonic vortices in the wSCS in summer have a more prominent impact on Chl-*a*, leading to lower concentrations of Chl-*a* (He et al., 2019). Another previous study found that the cold eddy water in wSCS contained higher nutrient salts, which could significantly improve the primary productivity of the sea area (Leng et al., 2016).

It is generally considered that directly sinking phytoplankton cells are major contributors to carbon export from surface layers, and it is an important part of ocean carbon sink (Boyd and Newton, 1999; Sun, 2011). It has been found that phytoplankton cells can regulate their sinking rate in a number of ways, such as their physiological state (Steele and Yentsch, 1960; Eppley et al., 1967), their morphology (Stokes, 1851; Kromkamp and Mur, 1984; Reynolds, 1987; Pitcher et al., 1989), light (Bienfang, 1985), and environmental factors such as temperature (Eppley, 1972) and nutrients (Titman and Kilham, 1976). At present, the SECTOL method described by Bienfang (1981) is generally accepted as the most accurate method to calculate the precipitation rate of phytoplankton, which can measure not only phytoplankton community sinking rate but also species-specific sinking rate (Pitcher et al., 1989; Peperzak et al., 2003; Pantorno et al., 2013). A recent study has shown that in the Changjiang (Yangtze River) estuary, a significant correlation was observed between phytoplankton sinking rate and phytoplankton community structures in the surface layer: the higher dominance of Bacillariophyta in the phytoplankton community corresponded to higher phytoplankton sinking rate (Guo et al., 2016). In the present study, in order to explore the influence of mesoscale physical processes on phytoplankton community structure and sinking rate in the wSCS, 64 initial water samples of phytoplankton, 192 sinking rate samples, and 576 Chl*-a* samples were measured in 16 stations. This study may yield a better understanding of the mesoscale physical processes on phytoplankton communities and sinking rate in the wSCS.

## Materials and Methods

### Study Area and Sampling Stations

**FIGURE 1:**
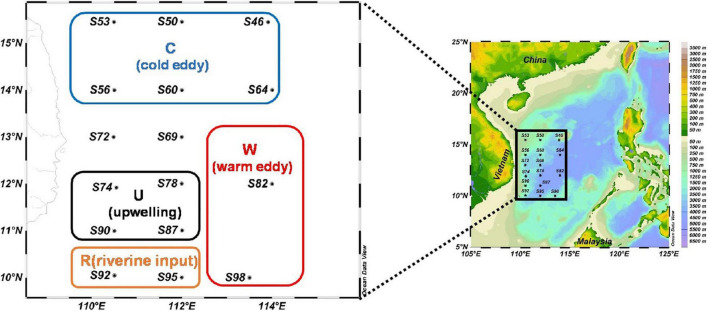
The investigation map in the western South China Sea (C: areas affected by cold eddy; W: areas affected by warm eddy; U: areas affected by upwelling; and R: areas affected by riverine input).

This research was carried out to determine phytoplankton community and sinking rate in the western part of the SCS (110.48–114.00 °E, 10.04–15.45 °N) from September 22 to October 11, 2016, by the RV “Experiment 3.” A total of 16 sample stations were used for sample collection. The sampling stations are shown in Figure 1. A variety of mesoscale physical processes (cold eddy, warm eddy, upwelling, and diluted water) occurred during the sampling, which had a great influence on the content of this study. According to where mesoscale physical processes occurred, this paper selects four regions (C: cold eddy, W: warm eddy, U: upwelling, and R: riverine input) that have significant influence on mesoscale physical processes.

### Sampling and Analysis

At each sampling station, the Seabird CTD was used to record the temperature, salinity, and fluorescence intensity of the seawater. Simultaneously, according to the fluorescence distribution, four layers (surface layer, maximum Chl*-a* layer, 100-m layer, and 200-m layer) were set, and 7-L seawater samples were collected from each layer, to determine Chl*-a* concentration, nutrient concentration, phytoplankton community, and phytoplankton sinking rate.

**FIGURE 2:**
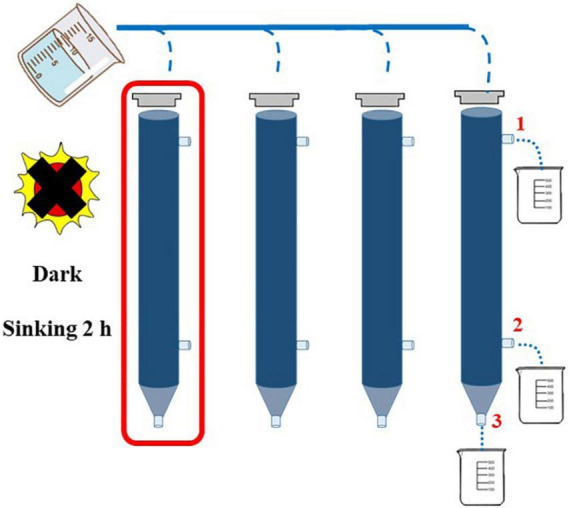
Application of SETCOL method in this study (Bienfang, 1981).

In this study, the concentration of Chl*-a* was determined by a fluorescence method (Welschmeyer, 1994). Two-liter seawater samples were filtered by GF/F film of 25 mm, and the filter film was kept under shading at −20^°^C. Chl*-a* was extracted in the dark with 90% acetone for 24 h at −20^°^C and measured with a Turner-Designs Trilogy TM fluorescence analyzer. Continuous Flow Auto Analyzer (Bran + Luebbe) was used to determine the concentration of nutrient such as NH_4_-N, NO_3_-N, PO_4_-P, SiO_3_-Si, and NO_2_-N in the laboratory (Liu et al., 2011). The structure of phytoplankton community was analyzed by the Utermöhl method (Sun et al., 2002). The identification of phytoplankton species taxa in the study area mainly referred to the article written by Sun et al. (2007). Cell volume conversion of phytoplankton was carried out according to the geometric model of cell volume (Sun and Liu, 2003). Carbon content and equivalent sphere diameter (ESD) of phytoplankton cells were converted according to cell volume (Eppley et al., 1970). The sinking rate of phytoplankton was determined by the SETCOL method (Bienfang, 1981). In order to acquire data with more credibility, four parallel water samples were set in each water layer, and each plexiglass column with a height of 0.53 m and a volume of 1,080 ml (Figure 2) was filled completely with a homogeneous seawater sample and capped. After sinking for 2 h, the water samples from one of the columns were collected in sequence from outlets 1, 2, and 3, the sinking rate of phytoplankton of each species was calculated in one column (red box), and the other three columns were used to calculate the total phytoplankton sinking rate.

### Data Analysis

The calculation formula of phytoplankton dominance (*Y*; Sun and Liu, 2004) is as follows:


(1) $$Y=\frac{n_{i}}{N}f_{i}$$

where *n*_*i*_ is the cell abundance of species *i* phytoplankton species in all samples, *N* is the cell abundance of all species, and *f*_*i*_ is the frequency of occurrence for species *i* in all samples.

The sinking rates of phytoplankton is calculated as follows (Bienfang, 1981):


(2) $$\psi =\left(B_{s}/B_{t}\right)\times L/t$$

where ψ is sinking rate, *B*_*s*_ is the biomass settled into the bottom compartment, *B*_*t*_ is the total biomass in the column, *L* is the height of the column, and *t* is the settling interval.

Phytoplankton cell carbon and ESD were calculated by the following formula (Eppley, 1972):


(3) $$Log_{10}C=0.76\times Log_{10}V-0.352\left(\text{for}\text{diatoms}\right)$$

(4) $$Log_{10}C=0.94\times Log_{10}V-0.60\left(\text{for}\text{other}\text{algae}\right)$$

(5) $$\mathrm{ESD}=2\times \sqrt[{3}]{\frac{3\times V}{4\times \mu }}$$

where *C* is the carbon of per cell (pg C cell^–1^) and *V* is the cell biovolume of each species (μm).

The carbon flux (*F*) of phytoplankton is calculated according to the following formula:


(6) $$F=\psi_{a}\times C_{a}$$

where *F* is phytoplankton carbon flux, ψ_*a*_ is the average of the sedimentation rate in water column, and *C*_*a*_ is the average of the phytoplankton biomass carbon.

In this study, Ocean Data View was used to draw and analyze the location map of the sampling station and the temperature and salinity distribution map. The Chl*-a* distribution and phytoplankton community composition were plotted and analyzed by Origin 2016. SPSS14.0 software was used to analyze the correlation between phytoplankton sedimentation rate and environmental factors.

## Results

### Hydrographic Conditions of the Survey Area

**FIGURE 3:**
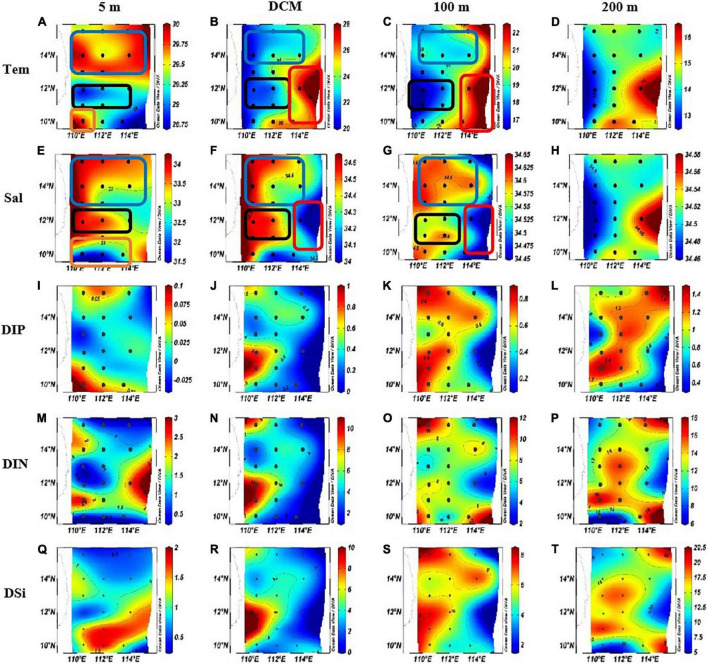
The horizontal distributions of temperature (Tem) (^°^C), Salinity (Sal) and nutrient concentration in the western South China Sea in autumn 2016. **(A)** Surface temperature. **(B)** DCM layer temperature. **(C)** 100 m layer temperature. **(D)** 200 m layer temperature. **(E)** Surface salinity. **(F)** DCM layer salinity. **(G)** 100 m layer salinity. **(H)** 200 m layer salinity. **(I)** surface DIP concentration. **(J)** DCM layer DIP concentration. **(K)** 100 m layer DIP concentration. **(L)** 200 m layer DIP concentration. **(M)** Surface DIN concentration. **(N)** DCM layer DIN concentration. **(O)** 100 m layer DIN concentration. **(P)** 200 m layer DIN concentration. **(Q)** Surface DSi concentration. **(R)** DCM layer DSi concentration. **(S)** 100 m layer DSi concentration. **(T)** 200 m layer DSi concentration.

The planar distribution of temperature, salinity, and nutrient (DIP: dissolved inorganic phosphorus; DIN: dissolved inorganic nitrogen; and DSi: dissolved inorganic silicate) at each water layer is shown in Figure 3. The temperature of the survey sea area ranged from 12.98 to 29.95^°^C (average = 21.38 ± 5.77^°^C; Figures 3A–D). The salinity ranged from 31.66 to 34.64 PSU (average = 34.20 ± 0.65 PSU; Figures 3E–H). In the north part of the survey area, an obvious area of low temperature and high salinity at shallow water above 100 m (blue box) proves that there had been a cold eddy in the survey area. In the middle of the survey sea area, the lowest temperature and the highest salinity of each water layer were found (blank box). Combined with the previous studies, it can be proved that it was an upwelling region (Wu and Li, 2003; Xie et al., 2003; Hu and Wang, 2016). In the eastern part of the sea area, an area of high temperature and low salinity was found between the DCM layer and the 100-m layer (red box), which proved that a warm eddy occurred here. At the bottom of the survey area, high temperature and low salt surface water appeared (orange box), indicating that this area was heavily affected by riverine input water.

The concentration of DIP, DIN, and DSi varied from 0 to 1.48 μmol L^–1^ (average = 0.52 ± 0.43 μmol L^–1^; Figures 3I–L), 0.26–17.98 μmol L^–1^ (average = 6.52 ± 4.90 μmol L^–1^; Figures 3M–P), and 0.44–20.44 μmol L^–1^ (average = 6.36 ± 5.58 μmol L^–1^; Figures 3Q–T), respectively. In addition to the surface layer, the concentration distribution of nutrients in other water layers is similar in response to mesoscale physical processes: in the cold eddy area and the upwelling area, the concentration of all kinds of nutrients was high; in the warm eddy region, the nutrient concentration was low. In the surface seawater, the DIP concentration was higher in the riverine input area and the cold eddy area, while the concentration in other areas was lower; DIN concentration was lower in the cold eddy area, the upwelling area, and the dilute water area; and the concentration of DSi was lower in the cold eddy region and the upwelling region, while it was higher in the warm eddy region.

### The Distribution of Chlorophyll *a* and the Phytoplankton Community Structure

Through microscopic examination of the samples in the wSCS in the autumn of 2016, a total of 108 phytoplankton species were identified belonging to 54 genera from the samples collected in the present investigation, including 50 species of Bacillariophyta, 54 species of Dinophyta, 3 species of Cyanophyta, and 1 species of Chrysophyta. Bacillariophyta and Dinophyta were the main phytoplankton communities in the investigated sea area, whose species number accounts for 96% of the total phytoplankton species. The dominant phytoplankton species in the sea area are shown in Table 1. The most dominant species was *Trichodesmium thiebautii*. After removing all cyanobacteria, the dominance of *Coscinodiscus argus* was significantly higher than other species, accounting for 31% of the total phytoplankton cell abundance.

**TABLE 1:** Dominant species in the western South China Sea in autumn 2016.

Species	Cell abundance ratio (%)	Dominance
*Trichodesmium thiebautii*	61.88	0.0967
*Coscinodiscus argus*	6.15	0.0423
*Thalassionema nitzschioides*	3.57	0.0340
*Thalassionema frauenfeldii*	3.38	0.0296
*Trichodesmium erythraeum*	18.11	0.0170
*Prorocentrum lenticulatum*	1.94	0.0169
*Thalassiothrix longissima*	0.51	0.0029
*Coscinodiscus subtilis*	0.48	0.0028
*Eunotogramma debile*	0.49	0.0021
*Prorocentrum compressum*	0.45	0.0021

**FIGURE 4:**
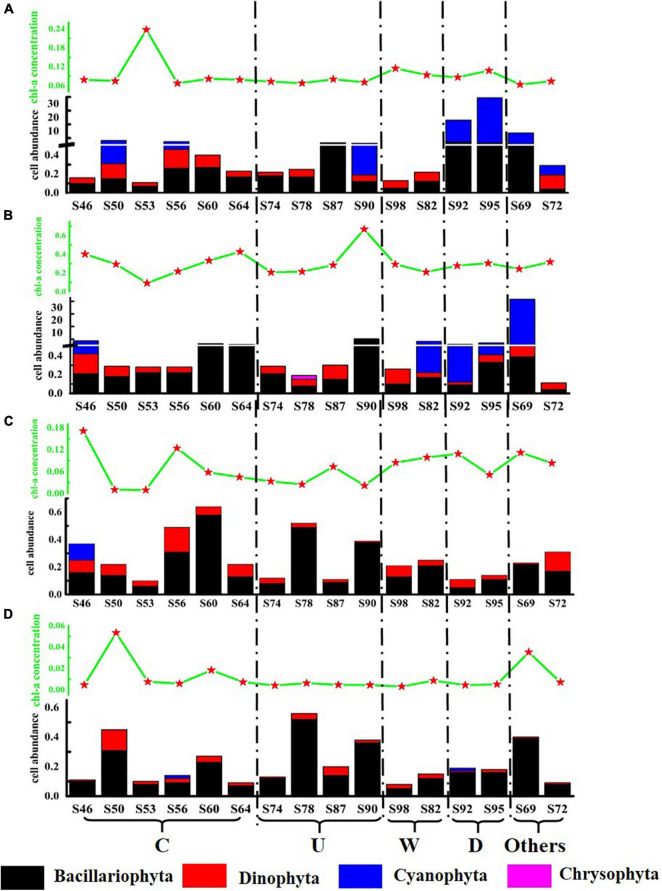
Schematic diagram of Chl*-a* concentration (μg L^–1^) and phytoplankton cell abundance (cell L^–1^) at the survey station in the western South China Sea in autumn 2016. **(A)** 5 m layer; **(B)** DCM layer; **(C)** 100 m layer; and **(D)** 200 m layer.

In order to intuitively compare the effects of mesoscale physical processes on phytoplankton abundance and community distribution, the stations were divided into five regions: region C affected by cold eddy (S46, S50, S53, S56, S60, and S64); region U affected by upwelling (S74, S78, S87, and S90); region W affected by warm eddy (S82 and S98); region R affected by riverine input (S92 and S95); and others that were not affected by mesoscale physical process. The concentration of Chl*-a* and the distribution of phytoplankton cell abundance at each station in the investigated sea area are shown in Figure 4. The variation range of Chl*-a* concentration was 0.003–0.666 μg L^–1^, with an average value of 0.117 μg L^–1^. The concentration of Chl*-a* has little correlation with the abundance of phytoplankton cells, which may be related to the different phytoplankton species in each water layer after the influence of mesoscale physical processes. The cell abundance of *T. thiebautii* had no significant contribution to the concentration of Chl*-a*. For example, in the surface layer of S95 and the DCM layer of S69, *T. thiebautii* account for 96.0 and 98.3% of the total cell abundance, respectively, but the concentration of Chl*-a* was below the other stations. The cell abundance of diatom directly affected the concentration of Chl*-a*. In this study, it was found that the concentration of Chl-*a* in the DCM layer was always high in the station with high abundance of *C. argus*, such as S90 and S64.

Both the cold eddy and upwelling brought the deep cold water to the upper layer. Because each station had a different effect, in this study, according to the distribution of temperature and salinity, the most influential stations were selected for analysis. The most affected station in the cold eddy area was S53, in which the cell abundance of diatom increased significantly. The most affected stations in the upwelling area were S90 and S87; the cell abundance of diatom was significantly increased. The rise in cell abundance of diatom is caused by the cold eddy and upwelling, in which the main species was *C. argus*. As a result of warm eddy, *T. thiebautii* in the surface layer was brought to the DCM layer at S82. The surface seawater of S92 and S95 was affected by fresh water, and the cell abundance was significantly higher than the other stations, among which *T. thiebautii* is greatly abundant. It was preliminarily concluded that the fresher water brought the extreme abundance of *T. thiebautii*.

### Carbon Biomass of Phytoplankton

**FIGURE 5:**
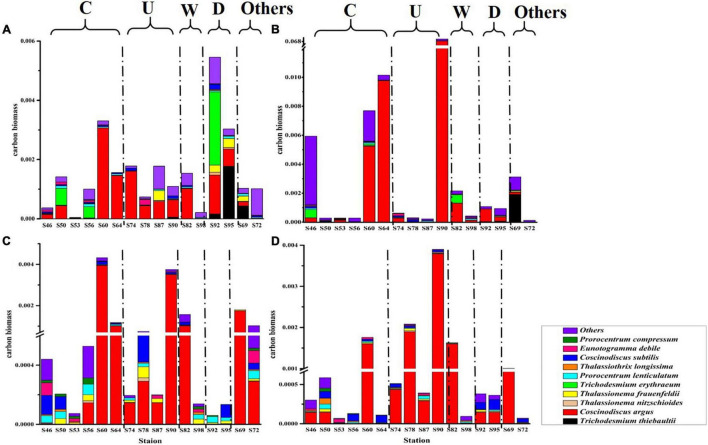
The carbon biomass (mg C L^–1^) of phytoplankton at the survey station in the western South China Sea in autumn 2016. **(A)** 5 m layer; **(B)** DCM layer; **(C)** 100 m layer; and **(D)** 200 m layer.

The carbon biomass of dominant phytoplankton species and other species in the survey area is shown in Figure 5. The variation range of phytoplankton carbon biomass was 4.36 × 10^–5^–0.07 mg C L^–1^, and the mean value was 0.002 mg C L^–1^. The largest phytoplankton carbon biomass was in the DCM layer, and most of the phytoplankton carbon biomass were contributed by *C. argus*. In region C and region U, it can be seen that *C. argus* has the least contribution to the carbon biomass in the surface layer and has a great contribution in the DCM layer and 100-m layer. According to the formula in *section “Sampling and analysis,”* the ESD of *C. argus* is about 58.294 μm. Compared with other dominant species, *C. argus* is larger in size and does not have characteristics that can resist sedimentation, so it can easily live in the DCM layer and deeper water layer. In this study, it was found that cold eddy and upwelling could bring the deep sea water that is rich in *C. argus* to the surface, and *C. argus* only stayed on the surface for a short time and then continued to sink to the DCM layer or deeper layers. Warm eddy brought phytoplankton from the surface to the deep water, leading to the increase of carbon biomass in the deep water. The riverine input brought a high abundance of cyanobacteria from the river to the ocean, leading to the highest carbon biomass in the surface water.

### Sinking Rate of Phytoplankton

**FIGURE 6:**
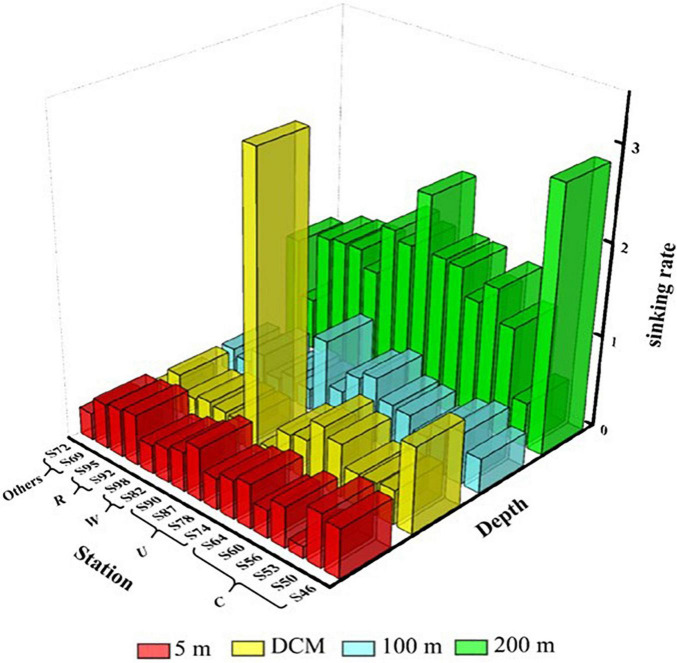
Sinking rate (m day^–1^) of phytoplankton at the survey station in the western South China Sea in autumn 2016.

The sinking rate of phytoplankton is shown in Figure 6. The range of the sinking rate of phytoplankton was 0.12–3.17 m day^–1^, and the average value was 0.72 m day^–1^. The sedimentation rate of phytoplankton was highest in the 200-m layer and relatively low in all other three water layers. The sedimentation rate of phytoplankton is governed by multiple factors. In this study, we found that various mesoscale physical processes in the marine area and the cell abundance of *C. argus* had the most significant effects on the sedimentation rate. What is more, different mesoscale physical processes affect the sedimentation rate by influencing the phytoplankton community distribution. The sinking rate of the DCM water layer at S60 and S64 in region C was relatively high, and the percentage of *C. argus* in diatom was 52.9 and 87%, respectively. Similarly, the sinking rate was the highest at S90 in region U, and the *C. argus* proportion of diatom in the DCM layer was 93%. The sinking rate of region W was relatively low in the surface layer to the 100-m layer, mainly because the warm eddy brought the surface sea water and active surface phytoplankton to the deep layer, which was caused by the upward movement of phytoplankton. The surface seawater of region R contains abundant phytoplankton, which were obtained from the riverine input, leading to the high sinking rate.

### Carbon Flux of Phytoplankton

**FIGURE 7:**
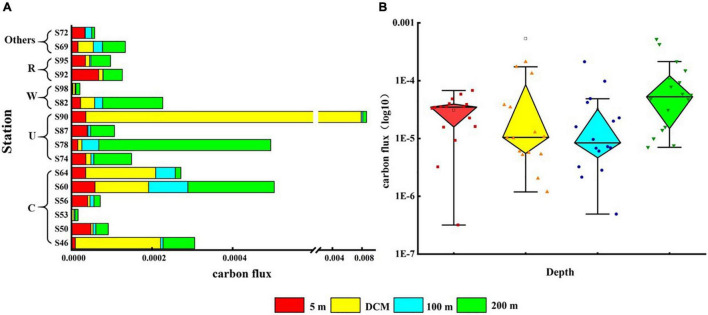
Carbon flux (mg C m^–2^ day^–1^) of phytoplankton at the survey station in the western South China Sea in autumn 2016. **(A)** Phytoplankton carbon flux in each water layer under various mesoscale physical processes; **(B)** summary of phytoplankton carbon flux at each station in four water layers.

The vertical distribution of phytoplankton carbon flux in the surveyed sea area is shown in Figure 7. The carbon flux of phytoplankton could directly represent the contribution of phytoplankton to carbon in each water layer. The variation range of phytoplankton carbon flux was 2.41 × 10^–6^–0.006 mg C m^–2^ day^–1^, and the average value was 0.0002 mg C m^–2^ day^–1^. Cold eddy and upwelling caused the carbon flux values of phytoplankton in the DCM layer to increase, with the most pronounced performance at station S90. Warm eddy caused lower carbon flux in all phytoplankton layers, as represented by station S98. The riverine input of fresh water caused the carbon flux of surface phytoplankton to increase, highlighted at station S92 (Figure 7A). Phytoplankton carbon flux was highest in the 200-m layer, followed by the DCM layer, and lowest in the 100-m layer (Figure 7B).

## Discussion

### The Sinking Rate of Phytoplankton and Its Controlling Environmental Factors

**FIGURE 8:**
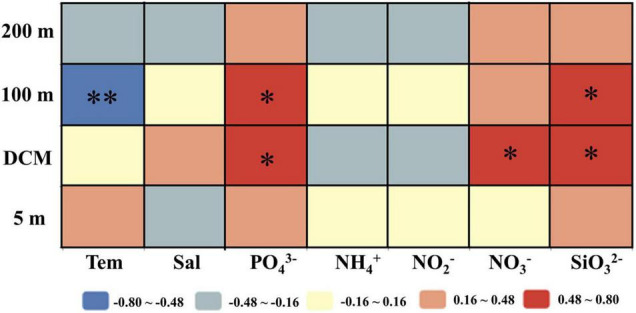
Relationships between phytoplankton sinking rate and environmental parameters in the survey area in the western South China Sea in autumn 2016. Pearson correlation coefficients (*r*) ranged from negative to positive and are indicated by color intensity changing from dark blue to red, respectively. ^**^p < 0.01; *p < 0.05 (two-tailed). Tem: Temperature; Sal: Salinity; PO_4_^3–^, NH_4_^+^, NO_2_^–^, NO_3_^–^, SiO_3_^2–^: concentration of each component.

The correlation analysis results between the sinking rate of phytoplankton and the environmental factors in each water layer of the investigated sea area are shown in Figure 8. It has been found that the sinking rate of phytoplankton measured in the laboratory was mainly determined by environmental parameters (Bienfang, 1984; Johnson and Smith, 1986; Muggli et al., 1996; Pantorno et al., 2013). Different from the previous research, the results of this study showed that the sinking rate of phytoplankton was not significantly correlated with most environmental factors especially nutrient concentrations, which was similar to the research results of Guo et al. (2016). The sinking rate of phytoplankton in the 100-m water layer had a significant negative correlation with temperature, while the partial nutrient concentration in the DCM layer and 100-m layer had a positive effect on the sinking rate. In laboratory experiments, phytoplankton lived in a stable hydrological environment, and the sinking rate was greatly affected by environmental factors. However, due to the interaction of various mesoscale physical processes, the environmental parameters in the investigated sea area were changeable, so the environmental parameters had little influence on phytoplankton in the field experiment.

### The Sinking Rate of Dominant Phytoplankton Species

**FIGURE 9:**
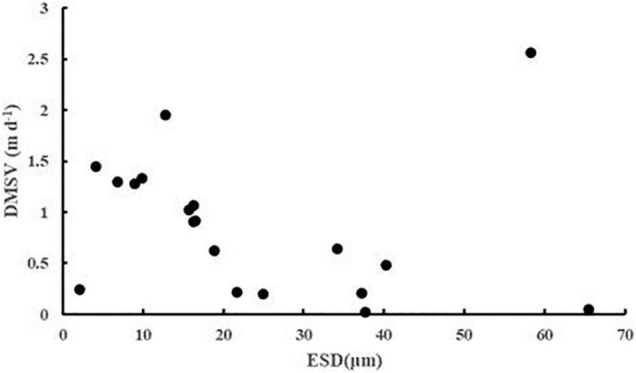
The ESD (μm) and DMSV (m day^–1^) scatter plots of the first 20 phytoplankton taxa in the survey area in the western South China Sea in autumn 2016.

The sinking rate of phytoplankton was affected by many factors. In order to explore whether cell morphology has a significant effect on the sinking rate of phytoplankton, the first 20 dominant species were selected to compare their cell ESD, direct measurement sinking velocity, and real specific vertical flux of a single species (Table 2). The sinking rate of phytoplankton was correlated with community structure (Culver and Smith, 1989). The sinking rate of Bacillariophyta was generally higher than that of Dinophyta, while that of Cyanophyta was the lowest. According to Stokes’ law, both cell size and cell density were important factors to determining the sinking rate. As shown in Figure 9, there was no significant correlation between the sinking rate of phytoplankton and its own cell size, which indicated that the cell density of phytoplankton had a greater influence on the sinking rate. Culver and Smith (1989) and Muggli et al. (1996) found that the sinking rate of phytoplankton cells was related to the physiological activity of the cells. Many studies had shown that the impact of water flow on phytoplankton was also extremely important. The scientists believed that water disturbance could reduce the precipitation rate of phytoplankton (Walsby and Reynolds, 1980; Lande and Wood, 1987); Other scientists suggested that water disturbance would accelerate the rate of phytoplankton deposition (Ruiz et al., 1996, 2004). Therefore, it is not enough to research the sinking rate of phytoplankton in the whole sea area by a single factor. More detailed studies on phytoplankton sinking rate should be carried out from various perspectives, such as phytoplankton cell density, cell physiological activity measurement, and sea water characteristics.

**TABLE 2:** The equivalent spherical diameter (ESD), direct measurement sinking velocity (DMSV), and real specific vertical flux (RSVF) of the first 20 phytoplankton taxa in the survey area in the western South China Sea in autumn 2016.

Species	Phylum	ESD (μm)	DMSV (m day^–1^)	RSVF (cell cm^–2^ day^–1^)
*Trichodesmium thiebautii*	Cyanophyta	37.61	0.02	–398.58
*Coscinodiscus argus*	Bacillariophyta	58.29	2.56	5, 250.72
*Thalassionema nitzschioides*	Bacillariophyta	6.79	1.30	1, 431.10
*Thalassionema frauenfeldii*	Bacillariophyta	12.84	1.95	1, 559.18
*Trichodesmium erythraeum*	Cyanophyta	65.48	0.05	2, 849.83
*Prorocentrum lenticulatum*	Dinophyta	16.25	0.91	399.36
*Thalassiothrix longissima*	Bacillariophyta	15.75	1.02	236.59
*Coscinodiscus subtilis*	Bacillariophyta	34.22	0.64	86.59
*Eunotogramma debile*	Bacillariophyta	24.97	0.20	–6.04
*Prorocentrum compressum*	Dinophyta	16.49	0.92	70.38
*Scrippsiella trochoidea*	Dinophyta	18.87	0.62	95.75
*Nitzschia* spp.	Bacillariophyta	4.12	1.45	186.25
*Pronoctiluca rostrata*	Dinophyta	16.25	1.07	62.11
*Thalassiosira minima*	Bacillariophyta	8.93	1.28	152.03
*Gymnodinium lohmannii*	Dinophyta	37.26	0.21	10.82
*Navicula* spp.	Bacillariophyta	9.85	1.33	86.98
*Coscinodiscus granii*	Bacillariophyta	40.24	0.48	45.99
*Richelia intracellularis*	Cyanophyta	2.11	0.24	107.60
*Prorocentrum lima*	Dinophyta	19.06	–0.03	–3.27
*Prorocentrum sigmoides*	Dinophyta	21.65	0.22	7.52

### Response of the Sinking Rate to Mesoscale Physical Processes

The SCS, which is located between the Indian monsoon and Asian monsoon region, is subject to seasonal risk control all year round, resulting in a multi-eddy structure (Wyrtki, 1961; Chen and Yang, 2010). Owing to the strong vertical stratification throughout the SCS, the growth of surface phytoplankton is limited by nutrient concentrations (Ning et al., 2004). Hence, the generation and reduction of numerous eddies can vary the amount of nutrients entering the euphotic layer and play an important role in the growth and distribution of phytoplankton (McGillicuddy et al., 2003; Lin et al., 2010; He et al., 2019). During the summer, an offshore rapid flow from the southwest to the northeast usually forms at about 12°N in the eastern part of Vietnam due to the influence of the southeast monsoon, which transports the cold water masses generated by the coastal upwelling to the basin area (Xie et al., 2003; Liang and Tang, 2017). The rapids are part of a cold–warm eddy dipole structure in the western part of the SCS during summer, located between the cold and warm eddies (Wang et al., 2006; Gan and Qu, 2008). The northern part of the rapids is frequently subjected to eruptive algal blooms into the basin, which explains the phytoplankton blooms in the western part of the SCS in summer (Tang et al., 2004a,b). The study of mesoscale physical processes has special significance for the development of ecology, chemistry, biology, and other disciplines. Therefore, mesoscale vortex plays a very important role in biogeochemistry.

**FIGURE 10:**
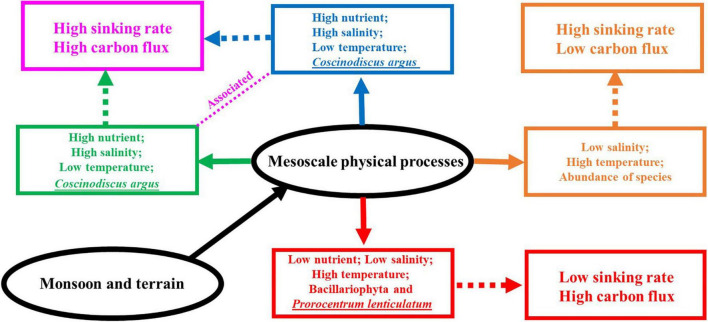
A conceptual schematic for the phytoplankton community and the sinking rate response to mesoscale physical processes (the blue solid line represents cold eddy, the green represents upwelling, the red represents warm eddy, and the orange represents riverine input water).

The correlation findings obtained in this study can be briefly summarized in conjunction with Figure 10. Due to the influence of cold eddy, the nutrient concentration of each water layer was relatively higher; furthermore, the concentration of Chl*-a* in the DCM layer was the highest at all stations except S53, which caused the maximum Chl*-a* layer to move up. The cell abundance of Bacillariophyta was larger in the DCM layer and the 100-m layer; *C. argus*, *Coscinodiscus subtilis*, *Thalassionema nitzschioides*, and *Thalassionema frauenfeldii* accounted for a large proportion, while Dinophyta was larger in the 5-m layer and DCM layer, mainly dominated by *Prorocentrum compressum* and *Prorocentrum lenticulatum*. The sinking rate of phytoplankton was higher in the 200-m layer and the DCM layer, but decreased in the 5-m layer. It is worth noting that the *C. argus* with high abundance appeared in both S60 and S64, and their sinking rates were very high in the DCM layer. Influenced by the cold eddy, a large number of *C. argus* were carried to the upper sea water, leading to a faster sinking rate of phytoplankton in the DCM layer. Surface phytoplankton were mainly *Prorocentrum* brought by cold eddy, and due to sufficient illumination and abundant nutrients, their physiological state was more active and thus the sinking rate was slower. In the area affected by upwelling, the nutrient concentration of each water layer increased, and the concentration of Chl*-a* was the highest in the DCM layer, followed by the 5-m layer. The cell abundance of Bacillariophyta at all stations except S87 showed an increasing trend with the deepening of water layer, in which the main species were *T. nitzschioides*, *T. frauenfeldii*, and *C. argus*. S87 showed the highest abundances in the 5-m layer; however, the dominant species were still *T. nitzschioides* and *T. frauenfeldii*. This is mainly due to the two species with high abundances at S87 that existed in single branches examined under the microscope, while at other stations, they existed in bunches or clusters; this form could not be easily carried to the upper seawater, such that the abundances increased in the deeper layers. The response to the upwelling was the most significant at S90, the *C. argus* cell abundance in this station was absolutely dominant, and the sinking rate was the highest at the DCM layer, reaching 3.17 m day^–1^. Generally speaking, the upwelling was associated with the cold eddy (Chai et al., 2001), and the high cell abundance of individual diatom species (such as *C. argus*) and the high phytoplankton sinking rate in the DCM layer in the study area were mainly affected by the cold eddy and the upwelling. It is inferred that there may be a connection between the abnormally high values of *C. argus* and the algal blooms formed by the rapids. In the area affected by the warm eddy, the concentration of nutrient in each water layer was decreased. The effect of warm eddy on Chl*-a* concentration was not obvious; the DCM layer was the largest, and the 200-m layer was the lowest. The cell abundance of Bacillariophyta at the 100-m layer was the largest, mainly of *T. nitzschioides* and *T. frauenfeldii*, while the cell abundance of Dinophyta at the 5-m layer and DCM layer was the largest, mainly of *P. lenticulatum*. Cyanophyta only appeared in the DCM layer. This means that warm eddy carried Bacillariophyta and Cyanophyta from the upper layer to the deeper water, and Dinophyta were still distributed in the upper layer because they could move by themselves. The influence of warm eddy on phytoplankton sinking rate was mainly reflected in the surface layer, while the values of sedimentation rate in the DCM and 100-m layer were lower and minimized in the DCM layer. This is mainly caused by the upward movement of phytoplankton as warm eddy carries the phytoplankton from the surface to deeper layers. Part of the sea area under investigation was affected by the fresh water, resulting in a high abundance of phytoplankton species and quantities in the surface layer and an increase in the number of dinoflagellate in the DCM layer, mainly *Prorocentrum* and *Gymnodinium*. The sinking rate in the surface layer was higher than that in the DCM layer and 100-m layer.

## Final Conclusion

There were many kinds of mesoscale vortices in the investigated sea area, and their effects on phytoplankton community and sinking rate were significant. The sinking rate of the DCM layer increased due to the cold vortex and the upwelling, and the response of *C. argus* was the largest. Warm eddy could reduce the sinking rate of phytoplankton in shallow water above 100 m. The carbon flux of phytoplankton showed that the bottom layer had the largest carbon flux, followed by the DCM layer, and the 100-m layer was the smallest, which could be well explained by the sinking rate of phytoplankton. The faster the sinking rate was, the greater the carbon flux contributed by phytoplankton. The division of mesoscale physical process regions in this study is mainly based on the *in situ* temperature and salinity data and the results of previous studies, but there is a lack of investigation and research related to the rapids in the sea area. Moreover, the sinking rate obtained in the experiment is only a relatively rough value, without considering the direction and velocity of the eddies in the sea area, which can still reflect the response of the phytoplankton community structure and sinking rate to the mesoscale physical processes. It was expected to provide basic data to study the response of phytoplankton sinking rate to mesoscale vortices in the SCS, and more accurate and specific studies are needed in the future.

## Data Availability Statement

The original contributions presented in the study are included in the article/supplementary material, further inquiries can be directed to the corresponding author/s.

## Author Contributions

JS designed the experiment. YM, YL, and GQ collected the samples. YM, XL, and YL performed the sample analysis. YM and JS wrote the manuscript, with contribution from all authors. All authors read and approved the final manuscript.

## Conflict of Interest

The authors declare that the research was conducted in the absence of any commercial or financial relationships that could be construed as a potential conflict of interest.

## Publisher’s Note

All claims expressed in this article are solely those of the authors and do not necessarily represent those of their affiliated organizations, or those of the publisher, the editors and the reviewers. Any product that may be evaluated in this article, or claim that may be made by its manufacturer, is not guaranteed or endorsed by the publisher.
